# Effects of temperature on plasma corticosterone in a native lizard

**DOI:** 10.1038/s41598-020-73354-z

**Published:** 2020-10-01

**Authors:** Andrea Racic, Catherine Tylan, Tracy Langkilde

**Affiliations:** 1 Department of Biology, The Pennsylvania State University, 208 Mueller Lab, University Park, PA 16802 USA; 2 Present Address: School of Dental Medicine, University of Pittsburgh, Pittsburgh, PA 15213 USA

**Keywords:** Ecology, Physiology

## Abstract

The glucocorticoid stress response is frequently used to indicate vertebrate response to the environment. Body temperature may affect glucocorticoid concentrations, particularly in ectotherms. We conducted lab manipulations and field measurements to test the effects of body temperature on plasma corticosterone (predominant glucocorticoid in reptiles) in eastern fence lizards (*Sceloporus undulatus*)*.* First, we acclimated lizards to one of 4 treatments: 22 °C, 29 °C, 33 °C, or 36 °C, and measured cloacal temperatures and plasma corticosterone concentrations at baseline and after exposure to a standardized stressor (cloth bag). Both baseline and stress-induced corticosterone concentrations were lower in lizards with lower body temperatures. Second, we acclimated lizards to 22 °C or 29 °C and exposed them to a standardized (cloth bag) stressor for 3 to 41 min. Lizards acclimated to 29 °C showed a robust increase in plasma corticosterone concentrations with restraint stress, but those at 22 °C showed no such increases in corticosterone concentrations. Third, we measured lizards upon capture from the field. There was no correlation between body temperature and baseline plasma corticosterone in field-caught lizards. These results suggest body temperature can significantly affect plasma corticosterone concentrations in reptiles, which may be of particular concern for experiments conducted under laboratory conditions but may not translate to the field.

## Introduction

Animals in the wild are constantly exposed to changing environmental conditions, including predator attacks^1,2^, fluctuating food availability^3^, and extreme and sudden weather abnormalities^4^. One common response to such stressors is the activation of the hypothalamic–pituitary–adrenal (HPA) axis, which results in the secretion of glucocorticoids to help the animal deal with the stressor and maintain homeostasis^5^. As a result, concentrations of glucocorticoids such as corticosterone or cortisol are often used as an indirect measure of stress^5–8^.

The glucocorticoid stress response, mediated by the HPA axis, is very similar in ectotherms such as reptiles to the series of events which occur in the HPA axis of mammals and birds^9^. In response to a stressor, the hypothalamus is stimulated to release corticotropin-releasing factor (CRF) and arginine vasopressin/vasotocin (AVP/AVT), depending on species^10^. CRF, and, to a lesser degree, AVP/AVT signal the release of adrenocorticotropic hormone (ACTH) from the corticotropic cells in the adenohypophysis of the pituitary gland^9,10^. ACTH then travels in the blood stream to the adrenal glands, where it stimulates the secretion of glucocorticoid hormones, the most important of which in reptiles is corticosterone (CORT)^9,10^.

Temperature affects many physiological processes, such as reproduction^11^, sprint speed and digestion^12^, and may similarly affect HPA functioning and thus CORT concentrations. Temperature affects CORT secretion in mammals; for example, at low body temperatures, sheep^13^ and dogs^14^ had a reduced stress response to physiological trauma as measured by plasma CORT concentrations. In both species, when body temperatures were returned to normal, CORT concentrations were restored, thus reversing the depressive effect of hypothermia with no lasting effects on HPA axis function^13,14^. However, since endotherms, like these dogs and sheep, have the ability to maintain a steady body temperature via internal mechanisms that release heat, body temperature is rarely considered when measuring hormone levels such as CORT^15^. However, body temperature plays a much larger role in the physiology of ectotherms^16^, as they are more susceptible to external influences on body temperature because of their limited metabolic control and reliance on behavioral thermoregulation^11^.

We examine the effects of temperature on the function of the HPA axis and subsequent secretion of plasma CORT in the ectothermic eastern fence lizard (*Sceloporus undulatus*). This species is a model for studying physiology^17–19^ and is widely used in studies exploring the effects of stressor exposure and CORT on physiology^20–22^, immunity^23–25^, survival and reproduction^26,27^, and offspring morphology^20,28^ in a laboratory environment—studies which are often performed at room temperature. Other studies performed in the field, when the animals are likely to be at their preferred body temperature, address the interactions between CORT and home range size^27^, novel stressors^29^, and seasonal effects^30^.

Eastern fence lizards are native to the southeastern United States, from southern New York to central Florida, west to eastern Kansas and central Texas^31^. This species exhibits seasonal variation in baseline plasma CORT concentrations, with higher CORT and sensitivity to ACTH during the spring breeding season versus the post-breeding season^27,32^. Their preferred body temperature in the field is 33 °C, maintained via thermoregulatory behavior such as basking^11^. Thermal preference in this species, as in many ectotherms, appears to be driven by optimization of physiological functions, with both locomotor and digestive functions optimized at this temperature^12^.

Yet information about the effects of temperature on CORT concentrations in this species, and in most reptile species, is scarce (but see^33–35^). Given how important maintaining body temperature within a narrow range is for other physiological processes^12^, it is likely equally important for the function of the HPA axis and subsequent release of both baseline and stress-induced CORT. Baseline plasma CORT concentrations in some reptile species are known to vary considerably between seasons^27,36^. This is often attributed to changes in reproductive activity throughout the year^27,36,37^, but unmeasured temperature changes between seasons may also contribute to this variation. Ambient temperature is rarely considered when discussing CORT concentrations in ectothermic species, but may be extremely important given its strong effect on body temperature and physiologic processes in ectotherms^38^. Such information is vital to understanding and interpreting the physiological effects of CORT on reptiles in laboratory studies.

Here we investigated whether (1) baseline and stress induced plasma CORT concentrations are affected by body temperature, (2) body temperature alters the increase in plasma CORT following exposure to a standardized stressor, and (3) body temperature of field-caught lizards correlates with their baseline plasma CORT concentrations. We hypothesize that baseline and stress-induced plasma CORT concentrations will be lower at cooler body temperatures, that CORT concentrations will not increase as much in response to a stressor at lower body temperatures, and that baseline plasma CORT concentrations of field-caught lizards will positively correlate to body temperature.

## Methods

### Ethics statement

The research presented here adheres to the Guidelines for the Use of Animals in Research and the Institutional Guidelines of the Pennsylvania State University. Approval for all procedures was granted by the Pennsylvania State University’s Institutional Animal Care and Use Committee (protocol number #44595-1). Animal collection was permitted by the respective states.

### Effects of body temperature on baseline and stress induced CORT

The lizards used in this study (n = 41; males = 19; females = 22) were the offspring of gravid females caught in Arkansas (St. Francis National Forest, n = 27) and Tennessee (Edgar Evins State Park, n = 5; Land Between the Lakes National Recreation Area, n = 9) in 2016 (see Ensminger et al.^20^, 2018 for details of adult female housing and egg incubation). After hatching, lizards were individually housed in plastic enclosures (31 × 26 × 20 cm, L × W × H) furnished with a shelter and paper towel substrate. The lizards were kept on a 12:12 h light:dark schedule, with an incandescent light bulb at one end of the enclosure to provide a temperature gradient for 8 h a day. Lizards were fed vitamin-dusted crickets (*Acheta domestica*) to satiation three times a week and water was available ad libitum.

For this experiment, lizards were assigned to one of 4 temperature groups (n = 10 or 11 per group), with approximately equal numbers of males and females in each group: 22 °C, 29 °C, 33 °C or 36°C^12^. These temperatures span the naturally experienced temperature range for this species. The coldest temperature, 22 °C, represents a cold morning in their natural range^31^ and approximates room temperature at which many experiments are run; 29 °C is a common daily temperature during their active period in the field^31^; 33 °C is the preferred body temperature of this species^11^*;* and 36 °C is close to the maximal temperature lizards experienced in the field while avoiding effects of heat stress^12^*.*

Lizards were kept in their individual home enclosures and the entire enclosure was placed in an incubator (130BLL, Percival Scientific, Inc., Perry, IA) set at the designated temperature (± 0.5 °C), with 4 enclosures per incubator. The lizards were allowed 4 h to acclimate to these conditions and for their body temperatures to reach the incubator temperature. The lizards were then removed from the incubator one at a time and their body temperature immediately measured via cloacal probe thermocouple attached to a thermometer (Fluke Thermocouple Thermometer 51/52 II). A blood sample was then obtained from their retro-orbital sinus using a heparinized capillary tube and transferred to a microcentrifuge tube. For each lizard, we recorded the time from first disturbance to the time at which blood sampling was completed (henceforth referred to as “time to bleed”). Lizards were then placed in a cloth bag, which served as a standard restraint stressor^29^, and returned to their home enclosure in the same incubator for 30 min. Lizards were then removed in their bags one at a time and a second blood sample obtained. Blood from both the baseline and post-stressor bleeds was kept on ice until all samples were obtained (2–4 h), spun at 3000×g for 5 min, and the plasma supernatant pipetted into a fresh microcentrifuge tube and stored at − 20 °C for later testing.

### Body temperature and CORT concentrations following exposure to an acute stressor

Adult lizards (n = 52; males = 30; females = 22) were captured from Kentucky (Land Between the Lakes National Recreation Area) in 2017, and individually housed in plastic enclosures (56 × 40 × 30 cm, L × W × H). Lizards were assigned to one of 2 temperature groups, with approximately equal numbers of males and females in each group: low (22 °C) or high temperature (29 °C) treatment. Lizards remained in their individual home enclosures and the entire enclosure was placed in a room set to the designated temperature (± 2 °C standard deviation) and allowed to acclimate to these conditions for 3 h. After acclimation, the lizards were placed in cloth bags to initiate an acute stress response^29^, and removed from the bags at randomly assigned times (3 to 41 min), at which time blood was obtained and body temperature measured, as described for the first experiment.

### Field body temperature and baseline CORT

Lizards (n = 98; males = 40; females = 58) were captured in April and May 2018 from sites in Alabama (Geneva State Forest, n = 15; Conecuh National Forest, n = 27; Blakely State Park, n = 16), Arkansas (St. Francis National Forest, n = 20), Kentucky (Land Between the Lakes National Recreation Area, n = 13), and Tennessee (Standing Stone State Park and Edgar Evins State Park, n = 7). All lizards were bled and their body temperature measured at the time of capture, as described in the first experiment.

### CORT assays

Plasma samples from the two laboratory studies were analyzed for CORT concentrations using the Corticosterone High Sensitivity EIA Kit (Immunodiagnostic Systems Ltd., Fountain Hills, AZ, USA). This kit was previously validated in this species^39^, and each sample was diluted 1:10 with assay buffer to ensure tested samples fell within the range of the standard curve. All samples were run in duplicate, with intra-assay coefficients of variation of 7.3–15.9 and an inter-assay coefficient of variation of 14.8.

Plasma samples from field-caught fence lizards were analyzed using the Corticosterone Enzyme Immunoassay Kit (Catalog number K014; Arbor Assays, Ann Arbor, MI, USA). Plasma was diluted 1:100 per manufacturer’s instructions, to ensure tested samples fell within the range of the standard curve. Percent recovery of CORT for this kit was approximately 95%, with good parallelism between the standard curve and a dilution curve made from pooled fence lizard plasma samples (CT unpubl. data). Each sample was run in duplicate, with intra-assay coefficients of variation of 5.6–9.2 and an inter-assay coefficient of variation of 6.3.

### Data analysis

Body weight and snout-vent length (SVL) were measured before each experiment (to the nearest 0.01 g and 1 mm, respectively), and body condition was calculated as the residuals of a linear regression of log-body weight to log-SVL. All treatment groups within each experiment had similar body condition mean and standard deviation. Logistical constraints resulted in different acclimation times for the two experiments conducted in the laboratory. This may have influenced the amount of CORT released, and to account for this we only compared lizards *within* each experiment; no comparisons were performed between experiments.

To assess the effects of body temperature on baseline and stress-induced CORT concentrations, we used a General Linear Model (GLM) with CORT concentration as a dependent variable, whether the CORT concentration was baseline or stress-induced as an independent factor (baseline vs. stress-induced bleeds), body temperature (representing the range of body temperatures produced after acclimating the lizards to their experimental temperatures) as independent continuous variables, and an interaction between body temperature and baseline versus stress-induced bleeds. Site was included as a factor. Incubator temperature was strongly correlated to cloacal temperature (r(81) = 0.82, *P* < 0.0001), thus was not included in the model to avoid multicollinearity issues. Cloacal temperature was included as a covariate in the model as it more precisely reflects the physiological effects of temperature on plasma CORT, as compared to incubator temperature. Sex, body condition, and time to bleed were initially included as covariates in the model. A model selection approach comparing Akaike Information Criterion values corrected for small sample sizes (AICc) to select variables for inclusion resulted in a best fit model which omitted sex, body condition and time to bleed. We forced the model to retain baseline versus stress-induced CORT, body temperature, an interaction between baseline versus stress-induced CORT and body temperature, and site since they were central to our hypotheses. CORT concentrations were log transformed to meet assumptions of normality.

To determine the effects of body temperature on CORT concentrations following exposure to a stressor, we used a GLM with plasma CORT as a dependent variable, room temperature (22 °C or 29 °C) as an independent factor, time exposed to an acute stressor (including all time spent in a cloth bag and the time taken to obtain blood sample after removal from the bag) as independent continuous variables, and an interaction between room temperature and time exposed to an acute stressor. Room temperature was strongly correlated to cloacal temperature (r(51) = 0.89, *P* < 0.0001), thus cloacal temperature was not included in the model to avoid multicollinearity issues. Room temperature was included as a covariate in the model as the intent of this experiment was to assess the effects of environmental temperature on stress-induced CORT release. Sex and body condition were initially included as covariates in the model. A model selection approach comparing Akaike Information Criterion values corrected for small sample sizes (AICc) to select variables for inclusion resulted in a best fit model which omitted sex and body condition. We forced the model to retain room temperature, time exposed to an acute stressor, and an interaction between room temperature and time exposed to an acute stressor since they were central to our hypotheses. Site was not included as a factor as all lizards in this experiment came from the same site. Plasma CORT concentration was log transformed to meet assumptions of normality.

We tested for an effect of body temperature on baseline plasma CORT concentrations in the field using a GLM with baseline plasma CORT of field-caught fence lizards as the dependent variable, and body temperature as the independent continuous variable. Site was included as a factor. Sex, time to bleed and body condition were initially included as covariates in the model. A model selection approach comparing Akaike Information Criterion values corrected for small sample sizes (AICc) to select variables for inclusion resulted in a best fit model which omitted sex, time to bleed, and body condition. We forced the model to retain body temperature and site since they were central to our hypotheses. Baseline plasma CORT concentration was log transformed to meet assumptions of normality.

All statistical analyses were performed using JMP (version 14.1.0, SAS Institute Inc., Cary NC) with α = 0.05. All analyses performed met assumptions of normality and homoscedasticity.

## Results

### Effects of body temperature on baseline and stress induced CORT

**Figure 1:**
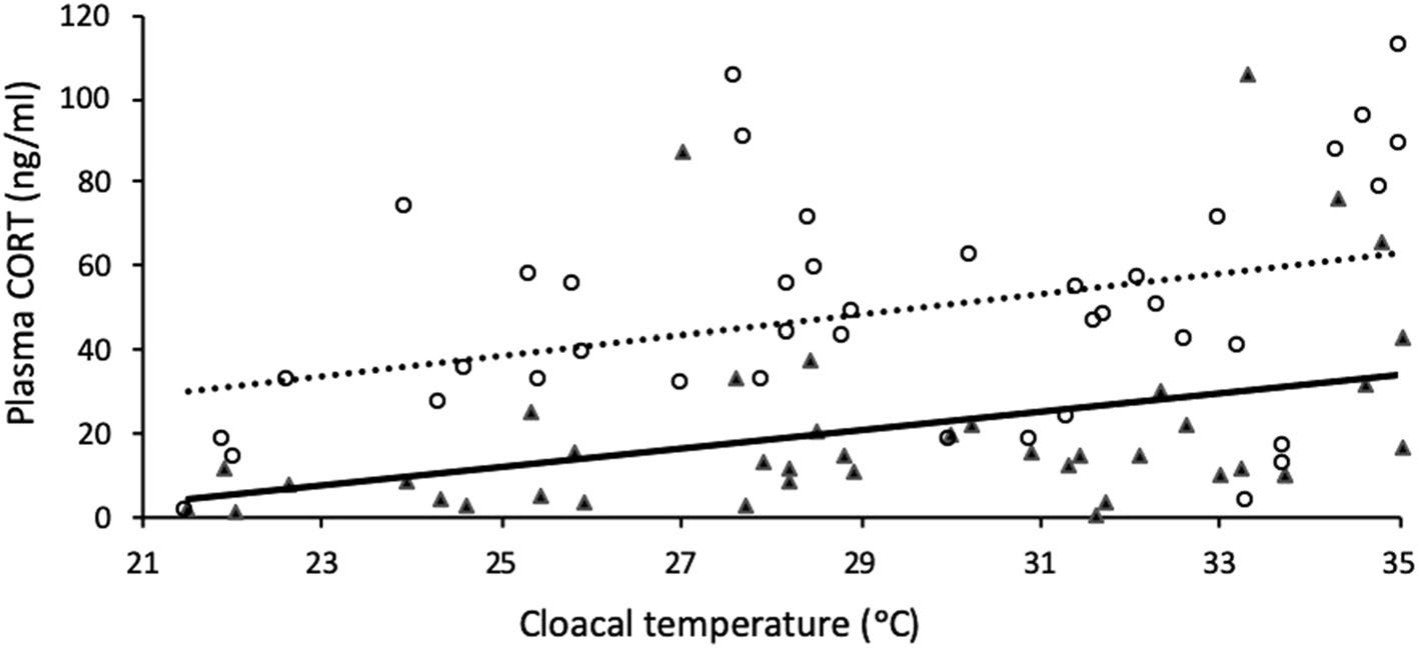
Baseline (solid line and triangles) and stress-induced (dotted line and open circles) plasma CORT concentrations of lizards (n = 41) at different body temperatures in the lab.

Baseline and stress-induced CORT concentrations were lower at lower body temperatures (F_1,76_: 9.26, *P* < 0.01; Fig. 1, Table 1). Stress-induced CORT concentrations were higher than baseline CORT concentrations (F_1,76_ = 31.15, *P* < 0.0001; Fig. 1, Table 1) but there was no interaction between baseline and stress-induced CORT concentrations and body temperature (F_1,76_ = 2.39, *P* = 0.13; Fig. 1). Baseline and stress-induced CORT concentrations did not vary by site of origin (F_2,76_ = 2.14, *P* = 0.12).

**Table 1:** Descriptive metrics taken for lizards from each study, mean ± standard error.

Experimental temperature (°C)	CORT (ng/mL)	Body (cloacal) temperature (°C)	Time to bleed (min)	Weight (g)	SVL (cm)
**Effects of body temperature on baseline and stress induced CORT**					
22	7.80 ± 2.22/33.54 ± 6.52^a^	23.74 ± 0.51	1.85 ± 0.08	6.04 ± 0.16	5.46 ± 0.05
29	24.66 ± 7.00/58.12 ± 6.96^a^	28.31 ± 0.51			
33	15.09 ± 2.75/42.41 ± 5.14^a^	30.97 ± 0.39			
36	38.53 ± 10.69/61.03 ± 12.31^a^	34.06 ± 0.24			
**Body temperature CORT concentrations following exposure to an acute stressor**					
22	11.44 ± 1.35	23.04 ± 0.10	3.60 to 40.90	13.17 ± 0.39	7.00 ± 0.06
29	14.74 ± 2.19	28.48 ± 0.25			
**Field body temperature and baseline CORT**					
–	13.62 ± 0.79	33.02 ± 0.29	4.11 ± 0.16	10.56 ± 0.34	6.49 ± 0.07

^a^Baseline plasma CORT concentrations/stress-induced CORT concentrations.

### Body temperature CORT concentrations following exposure to an acute stressor

**Figure 2:**
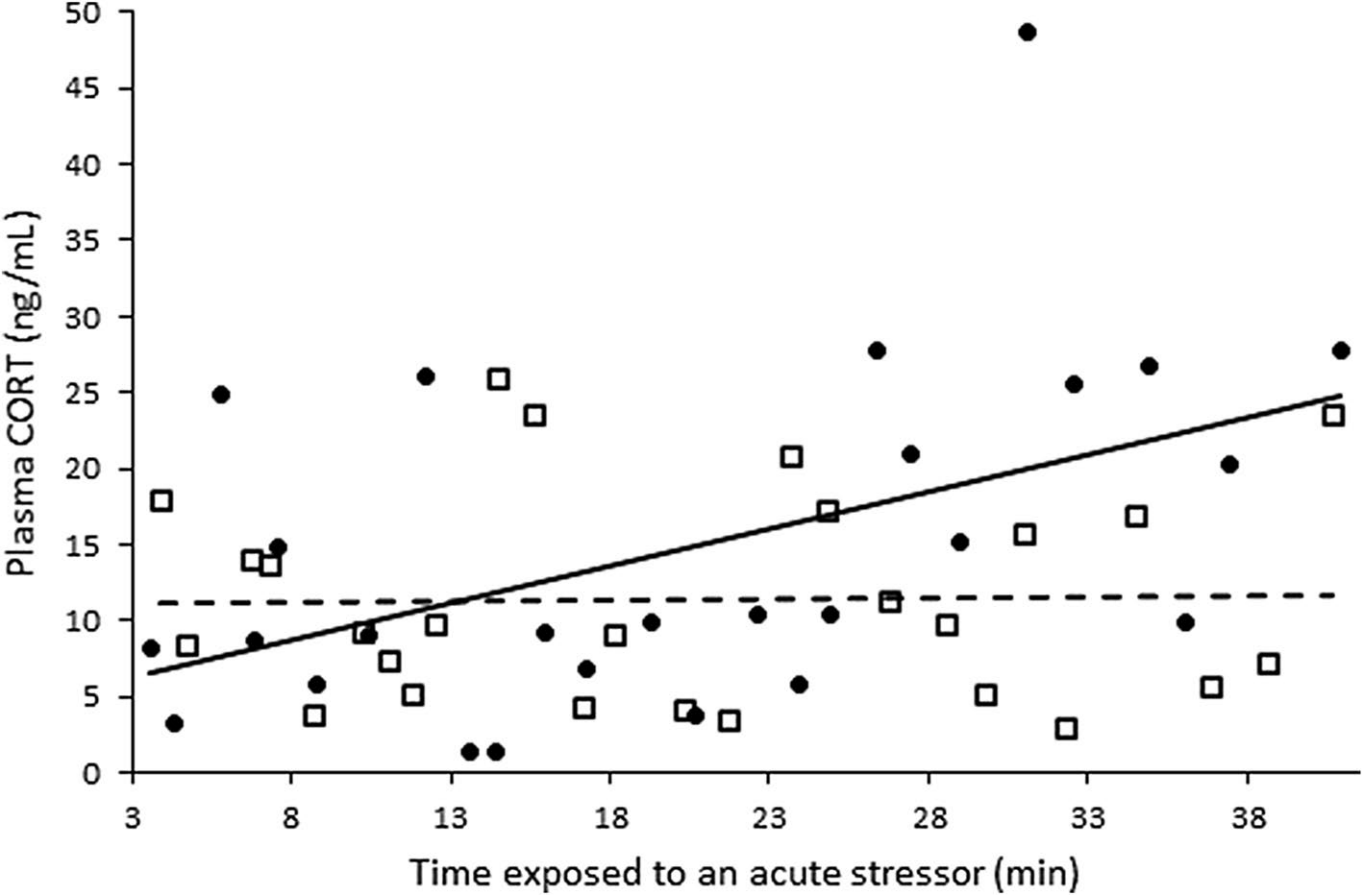
Plasma CORT concentrations of lizards following exposure to a stressor for different lengths of time (encompassing both time in a cloth bag and the time required to obtain blood) in a warm (solid line and circles) or a cool room (dashed line and open squares).

Room temperature did not affect plasma CORT concentrations overall (F_1,48_ = 0.37, *P* = 0. 55; Fig. 2, Table 1). Longer times exposed to an acute stressor (encompassing both time in the stress-inducing cloth bag and time taken to obtain blood) were not associated with increased plasma CORT concentrations overall (F_1,48_ = 0.01, *P* = 0. 93; Fig. 2). However, there was an interaction between room temperature and time exposed to a stressor in affecting CORT concentrations; lizards in the low temperature room (22 °C) did not show an increase in plasma CORT with increasing time exposed to a stressor, whereas lizards in the higher temperature room (29 °C) exhibited increased plasma CORT with longer time in the cloth bag (F_1,48_ = 4.45, *P* = 0.04, Fig. 2).

### Field body temperature and baseline CORT

**Figure 3:**
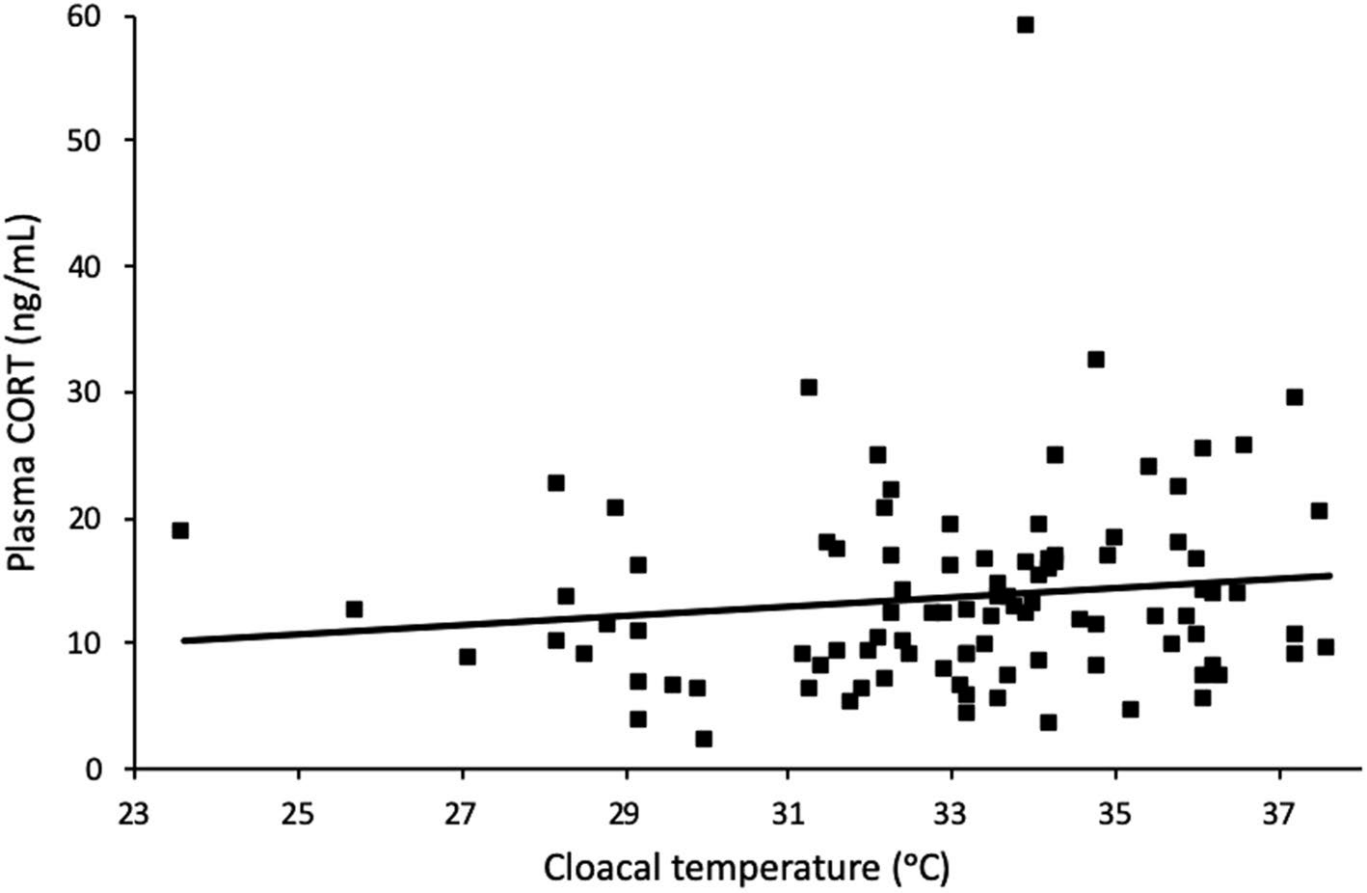
Baseline plasma CORT concentrations versus body temperature of lizards measured at the time of capture from the field.

Baseline plasma CORT concentrations in the field were unaffected by body temperature (F_1,91_ = 2.13, *P* = 0. 15; Fig. 3, Table 1) , but varied between capture sites (F_5,91_ = 3.45, *P* < 0.01).

## Discussion

Body (cloacal) temperature has a marked effect on plasma CORT concentrations in fence lizards. As hypothesized, we found that baseline and stress-induced plasma CORT concentrations were lower at cooler body temperatures. This effect is strong enough to confound baseline and stress-induced CORT concentrations if blood is taken at markedly different temperatures, as stress-induced CORT concentrations at lower body temperatures are similar to baseline CORT concentrations at higher body temperatures. Our hypothesis that lower temperatures would reduce stress-induced CORT concentrations was also supported, with the lower room temperature shown to completely suppress the increase in plasma CORT stimulated by a standard restraint stress protocol. However, our hypothesis that baseline plasma CORT would positively correlate to body temperature in the field was not supported by our data; there did not appear to be a significant effect of body temperature on baseline CORT in field-caught lizards. This could have important implications for lab experiments, the results of which could be affected if conducted at room temperature where lizards are unable to thermoregulate, but may not affect field studies where lizards are able to maintain body temperatures within physiologically-functional ranges^12^.

The primary lesson to be taken from this study is the need to account for the potential confounding effects of temperature when interpreting plasma CORT concentrations in ectotherms such as the eastern fence lizard. Conducting an experiment in the laboratory at room temperature is likely to produce very different baseline and stress-induced plasma CORT concentrations than the same experiment conducted in the field, or on a cool day versus a hot day, due in part to blunting of the CORT response at cooler body temperatures. For example, exposing fence lizards to attack by fire ants in the field is known to cause a marked glucocorticoid stress response^40^, but when performed in a laboratory environment at room temperature this effect may be blunted or absent^22^. This is well illustrated by the stress-induced plasma CORT concentrations of lizards acclimated to room temperature (22 °C), which were the same as the baseline CORT concentrations in lizards acclimated to warm field temperature (36 °C) (see Fig. 1). Temperature effects on the HPA axis may be particularly important when assessing stress-induced CORT, as lizards kept at room temperature completely failed to show an increase in CORT in response to a standard restraint stress protocol (see Fig. 2). This is consistent with studies in other reptile species, which show a positive correlation between higher temperatures and CORT concentrations after a standardized stress protocol in rattlesnakes^41^ and boa constrictors^42^. A likely sequela of this is that investigators may unthinkingly run restraint-stress, adrenocorticotropic hormone (ACTH) stimulation, or other stress protocols on ectotherms at room temperature, and then erroneously report no CORT increase in response.

This effect of body temperature on CORT could have important implications for field work, for example when measuring CORT at different times of the day or different days. However, body temperature did not appear to play a significant role in the baseline plasma CORT concentrations of field-caught fence lizards. This could be due to the fact that in the field lizards are able to thermoregulate, maintaining their body temperatures within a narrow optimal range^12^ and may thus be within the thermal range allowing appropriate CORT secretion. Most of the field-caught lizards in this study were measured during their prime activity period in the middle of the day, but lizards captured early in the morning, in the early part of their active period (from 0830 to 1630)^31^, or during an unseasonably cool or warm time may increase the variation in body temperatures seen (Langkilde, personal observations), which could result in detectable effects on CORT concentrations. It is also possible that lizard body temperature, even within the range observed in the field, may affect the stress-induced plasma CORT concentrations, which we did not measure. For example, rattlesnakes and boa constrictors show no correlation between body temperature and baseline plasma CORT, but a positive correlation between body temperature and stress-induced CORT^37,41^. This may indicate that the stress-response is more sensitive to differences in body temperature, and may be affected by temperature even when baseline CORT is unaffected. Additionally, at extreme temperatures far outside their preferred range, fence lizards may experience an increase in CORT due to activation of the stress response and HPA axis in order to adapt to the stresses of extreme temperatures, as has been shown in lizards subjected to temperatures near their thermal maximum (43 °C)^22^. Such an increase in CORT would be different from the increases due to temperature shown in this study, as it would be stimulated by a need to adapt to a temperature stressor rather than the elevated physiological function due to increased temperature measured here^22^.

Due to the widespread use of plasma CORT concentrations as a measure of stress, it is vital that it be interpreted correctly. Temperature is an important factor to account for, particularly when measuring CORT in ectotherms, whose body temperature can be influenced by the environment. It may be especially important for laboratory studies which for logistical reasons are sometimes conducted at room temperature, or in animals for which thermoregulation may be limited in captivity. All treatment groups should ideally be run at the same temperature within the optimum temperature range for the species, or body temperature should at least be measured and included in statistical models. This may be particularly crucial for studies involving stress-induced CORT concentrations, as the increase in CORT may be completely abolished at lower temperatures. Studies assessing the increase in CORT following a potential stressor may thus erroneously report no increase in CORT if the experiment is performed at room temperature. The effects of temperature on plasma CORT are profound enough to corrupt the results of otherwise well-designed studies and must be accounted for when designing and interpreting any study for which CORT is measured.

## Data Availability

The datasets generated during and/or analyzed during the current study are available from the corresponding author upon reasonable request.
